# Effects of Hematopoietic Cell Transplantation on the Pulmonary Immune Response to Infection

**DOI:** 10.3389/fped.2021.634566

**Published:** 2021-01-26

**Authors:** Matt S. Zinter, Janet R. Hume

**Affiliations:** ^1^Department of Pediatrics, Divisions of Critical Care and Bone Marrow Transplantation, University of California, San Francisco, San Francisco, CA, United States; ^2^Department of Pediatrics, Division of Critical Care Medicine, University of Minnesota Medical School, Minnesota, MN, United States

**Keywords:** hematopoietic stem cell transplant, immune response, invasive aspergillosis, pneumonia, pneumonitis, lung, pulmonary immunity

## Abstract

Pulmonary infections are common in hematopoietic cell transplant (HCT) patients of all ages and are associated with high levels of morbidity and mortality. Bacterial, viral, fungal, and parasitic pathogens are all represented as causes of infection. The lung mounts a complex immune response to infection and this response is significantly affected by the pre-HCT conditioning regimen, graft characteristics, and ongoing immunomodulatory therapy. We review the published literature, including animal models as well as human data, to describe what is known about the pulmonary immune response to infection in HCT recipients. Studies have focused on the pulmonary immune response to Aspergillus fumigatus, gram-positive and gram-negative bacteria, and viruses, and show a range of defects associated with both the innate and adaptive immune responses after HCT. There are still many open areas for research, to delineate novel therapeutic targets for pulmonary infections as well as to explore linkages to non-infectious inflammatory lung conditions.

## Introduction

Hematopoietic cell transplantation (HCT) is being used for treatment of an increasing number of malignant and non-malignant disorders in all age groups, but is associated with numerous complications. Infections are a major complication due to immune system derangements associated with HCT. In particular, pulmonary infections make up a large portion of HCT-related infections in both autologous and allogeneic HCT patients and are associated with significant morbidity and mortality (1, 2). Pulmonary infections in HCT patients are caused by a range of bacteria, viruses, fungi, and protozoal agents (2, 3). The most common pathogens change through time post-transplant as the immune function of the patient slowly recovers (2, 3). The pulmonary immune response after HCT is not as well-documented as the systemic immune response. We examine the literature examining pulmonary immune responses to infection after HCT and find documentation in defects in both the innate and adaptive immune responses to pulmonary infection with a variety of pathogens.

## Pulmonary Immunity and Hematopoietic Cell Transplant

The lungs are constantly exposed to a variety of pathogens and thus have a complex system of immune responses. The mucociliary elevator provides a first line of defense to trap and remove infectious agents. Resident alveolar macrophages (AMs) play an important role in initial recognition of pathogens *via* the innate immune response, using pattern recognition receptors (PRRs) that identify pathogen-associated molecular patterns (PAMPs) such as bacterial or fungal cell wall components or viral nucleic acids. Alveolar macrophages also have a critical function in activating immune and inflammatory responses. Airway and alveolar epithelial cells are increasingly recognized to have an important part in recognizing pathogens as well. On recognition of pathogens, production of inflammatory cytokines and chemokines leads to the recruitment of neutrophils and monocytes to the lung for phagocytosis and killing of pathogens. Antigen-presenting cells that include AMs as well as resident dendritic cells provide a bridge to activate the adaptive immune system by traveling to local lymphoid tissue and presenting antigens to T cells. The activation of T cell and B cell responses results in killing of intracellular pathogens, production of antibodies, development of immune memory, and regulation of the immune response. The T helper (Th) response can be polarized toward different pathways, such as Th1, Th2, Th17, and T regulatory (Treg), with varying effects on the predominant immune response (4–6).

Immune reconstitution post-HCT is a complex and prolonged process, with the timing affected by many factors including type of transplant (autologous vs. allogeneic), conditioning regimen (myeloablative vs. non-myeloablative), source of transplant (peripheral blood stem cells, bone marrow cells, umbilical cord blood stem cells), and presence of graft vs. host disease (GVHD) (7, 8). The appearance of innate immune cells such as neutrophils, monocytes, dendritic cells and NK cells in peripheral blood within the first few weeks after HCT is the first sign of immune reconstitution. T and B lymphocytes appear later in peripheral blood, but their function has been reported to be abnormal for months to years post HCT (9). As most of the scientific work has focused on numbers and function of immune cells circulating in the blood, we emphasize specifically the available literature on pulmonary immune function and reconstitution after HCT.

Overall, ample data suggest that recipient-derived AMs are only fully ablated with the most severe total body irradiation (TBI)-based conditioning, and for patients not receiving TBI-based conditioning, reconstitution of AMs from the donor lineage can take weeks. In mice, Matute-Bello et al. found that reconstitution of AMs of donor origin was slower than reconstitution of peripheral leukocytes after total body irradiation and syngeneic HCT (10). At 30 days post-transplant, 87.8 ± 3.9% of peripheral leukocytes were of donor origin, but AMs were only 74.5 ± 2.4% donor origin at 60 days post HCT (10). A comparison of different TBI and chemotherapy regimens for pre-syngeneic HCT conditioning found that TBI-based conditioning resulted in the highest rate of reconstitution of AMs with donor cells making up 82 ± 2% of AMs at 5 weeks after TBI (11). In contrast, splenic cells had higher percentage of donor derived cells (95 ± 1%) (11). TBI-sparing chemotherapeutic regimens proved to be much less effective in reconstitution of AMs with donor cells, with only 56 ± 6.2% of AMs of donor origin at 5 weeks post-HCT with a combined cyclophosphamide and busulfan regimen (11). Hahn et al. examined AM repopulation after TBI and also found a slow repopulation of alveolar and lung parenchymal macrophages, requiring 10 weeks post-transplant to reach ~90% donor derived cells (as opposed to 14 days for peripheral leukocytes) (12).

However, lung dendritic cells were much more sensitive to radiation, with more than 90% of CD103^+^ DCs depleted by day 3 post-irradiation and repleted with more than 90% donor cells by day 7 post irradiation. CD11b^+^ DCs were less sensitive, with 60% depletion by day 5 post-transplant and reconstitution with more than 90% donor derived cells at 4 weeks post-transplant (12). In humans, analyses of BAL fluid post HCT showed a significant increase in the number of AMs starting only at day 50 post HCT. The AMs after day 50 also had increased expression of markers of proliferation and appeared to be mostly of donor origin (13). Another study on BAL fluid post HCT showed reconstitution of AMs by cells of donor origin by ~90 days (14). These studies demonstrate that reconstitution of immune cells in the lungs proceeds at a slower pace than in peripheral blood, and that there are differences in reconstitution among different cell populations.

While donor-derived alveolar epithelial cell reconstitution appears sparse in all but the harshest TBI-based conditioning (10, 15), patient-derived alveolar epithelial cells, which typically repopulate from local progenitors, experience a variety of post-HCT insults including cellular atypia, altered cytokine production, and loss of surfactant biosynthesis (6, 16). Data on the efficacy and kinetics of ablation and repopulation of other lung-specific cells such as resident memory T-cells are lacking at this time.

Studies have described functional defects post-HCT in both innate and adaptive immune responses in the lungs, against a variety of pathogens. The majority of functional studies have involved mouse models of HCT, the majority of which involve syngeneic transplants. There are some studies of human HCT patient bronchoalveolar lavage (BAL) and lung tissue that provide information on immune response in the human lung. In addition, studies of genetic polymorphisms predisposing to infection in human HCT patients have provided suggestions of the importance of particular components of the immune response to the response to pulmonary infections in HCT patients. The primary areas of study have focused on the following three pathogens or groups of pathogens: Aspergillus fumigatus, gram-negative and gram-positive bacteria, and viruses (primarily herpesviruses).

## Pulmonary Aspergillosis

A substantial body of work on pulmonary immune responses to infection after HCT has focused on the ubiquitous mold Aspergillus fumigatus. Invasive aspergillosis (IA) most commonly involves the lung, is the most common invasive fungal infection in HCT patients, and has reported case fatality rates of 29–87% (1, 17). Immune defense against A. fumigatus requires both innate and adaptive immune responses, and deficiencies in both arms of the immune response have been associated with increased vulnerability to and defective response to IA (17).

Detection and first response to Aspergillus *via* the innate immune response and antigen presenting cells is critical to anti-fungal immunity. The importance of PRRs in activating the innate response to IA is suggested by a number of studies associating polymorphisms in human PRRs with susceptibility to IA in HCT patients. Toll-like receptors (TLRs) comprise a family of PRRs that play a key role in the innate immune response by recognizing a range of PAMPs and signaling to produce a pro-inflammatory response. Donor-derived polymorphisms in TLR1, TLR4, and TLR6 have been associated with increased risk of IA in HCT patients (18–20). Conversely, Carvalho et al. found an association of a donor-derived TLR4 polymorphism with increased risk of fungal colonization but decreased risk of fungal infection (primarily caused by Aspergillus spp.) (21). These studies did not address the mechanisms by which the polymorphisms would cause increased risk of IA but emphasize the general importance of the transplanted innate immune system's ability to recognize and respond to Aspergillus antigens.

A polymorphism in another PRR important in recognition of A. fumigatus, the C-type lectin Dectin-1, which results in an early stop codon and decreased Dectin-1 activity, increased the susceptibility to IA when present in either the donor or recipient of allogeneic HCT (22). In a mouse allogeneic HCT model of IA, knocking out Dectin-1 had varying effects depending on whether the knockout was present in the donor or recipient. Recipient mice with Dectin-1 KO had worsened control of fungal infection and increased inflammatory response in the lungs characterized by increased production of IL-17, with the most severe phenotype seen in Dectin-1 KO mice transplanted with Dectin-1 KO hematopoietic cells. However, recipient Dectin-1^+^ mice transplanted with Dectin-1 KO hematopoetic cells had fungal susceptibility equivalent to WT mice in the setting of a cytokine response dominated by IFN-γ and IL-10. This suggests a complex role for Dectin-1 in immune defense against A. fumigatus, with Dectin-1 on non-hematopoietic (presumably epithelial) cells providing control of infection *via* IFN-γ and IL-10 production and Dectin-1 on hematopoietic cells driving an IL-17 mediated response (22).

The intracellular PRR NOD-2 has also been found to have a polymorphism that, when present in the donor, significantly increased the risk of IA in allogeneic HCT recipients (23). Patients with the at-risk polymorphism and IA also had significantly increased levels of IL-8 and IL-10 in BAL fluid relative to IA patients without the polymorphism. In addition, peripheral blood mononuclear cells from patients with the at-risk polymorphism produced significantly less IL-1β *in vitro* in response to A. fumigatus. In a cyclophosphamide treated mouse model, NOD-2 KO mice had better survival and decreased fungal burden and lung and sinus inflammation after A. fumigatus infection (23). These results suggest that NOD-2 contributes to an inflammatory response to IA that may be detrimental.

The secreted PRR long pentraxin 3 (PTX3), which acts as an opsonin for A. fumigatus, was described to have a donor-derived polymorphism associated with increased IA risk in allogeneic HCT patients. Individuals who received a transplant with this haplotype had decreased production of PTX3 in BAL fluid and in lung tissue in the setting of IA. Neutrophils from patients with the polymorphism demonstrated reduced phagocytosis and killing of A.fumigatus that was restored by exogenous PTX3 (24).

Defects in anti-A. fumigatus immunity due to impaired cellular killing have also been identified. Effective clearance of A. fumigatus with appropriate control of the inflammatory response involves an autophagy process known as LC3-associated phagocytosis. IFN-γ activates a signaling response that includes death-associated protein kinase 1 (DAPK1). A polymorphism in patient-derived DAPK1 was associated with a significantly increased risk of IA in allogeneic HCT recipients. HCT recipients with the at-risk polymorphism in DAPK1 also demonstrated decreased expression of DAPK1 and increased expression of the inflammasome component NLRP3 and production of IL-1β in PBMCs in response to A. fumigatus conidia, consistent with a phenotype of increased inflammation (25).

Alterations in antigen presentation and the activation of the adaptive immune system have also been shown to affect susceptibility to IA. The chemokine receptor CCR7 has a key role in driving migration of DCs from sites of infection to secondary lymphoid organs *via* its ligands CCL19 and CCL2. Hartigan et al. demonstrated, unexpectedly, that CCR7 deficient mice had improved survival and decreased lung inflammation compared to WT mice in a neutropenic model. Neutropenic mice receiving syngeneic HCT of CCR7 KO cells also had improved survival and decreased inflammation in Aspergillus infection, associated with increased dendritic cell recruitment to the lung (26). These results suggest that CCR7-mediated responses in DCs may be associated with an excessively pro-inflammatory response that has detrimental effects on survival.

Further evidence for the importance of dendritic cells in controlling the infection and inflammatory response in IA came in a model of syngeneic HCT where mice underwent adoptive transfer with DC pulsed with A. fumigatus conidia prior to pulmonary A. fumigatus infection. When transferred DCs were primed *via* the TLR9 pathway with thymosin α1 prior to transfer, there was significantly improved survival, control of fungal replication, and decreased pulmonary inflammation in mice with IA. The thymosin α1-primed DCs stimulated a Th1/Treg response, suggesting this type of T cell polarization is important for successful defense against IA (27).

Epithelial cells also have an important role in recognizing pathogens and activating the immune response (5). De Luca et al. showed in a allogeneic mouse HCT model that lung epithelial cell response to A. fumigatus is mediated through the Toll-like receptor 3/Toll/IL-1 receptor domain-containing adaptor-inducing interferon (TLR3/TRIF)-dependent pathway (28). TRIF deficiency in either donor or recipient was associated with increased fungal growth and pulmonary inflammation associated with upregulation of IL-17 and downregulation of IFN-γ and IL-10. These findings suggest that a Th1/Treg response, *via* a TRIF-dependent pathway, is necessary for effective clearance and controlled inflammatory response to IA, while a Th17 type response is detrimental for the response to IA (28).

Finally, further suggestion that Th polarization is important in the outcome of IA in HCT patients comes from studies of polymorphisms in IL-17 and IL-23 genes, which are key in mediating the Th17 pathway. A patient-derived polymorphism in the IL-23A gene was identified to be associated with decreased risk of fungal infection (primarily Aspergillus spp.) in a group of allogeneic HCT recipients (29). Taken together, these data indicate a significant role for impaired A. fumigatus immunity post-HCT through impairments in PRR detection, phagocytosis mechanisms, cytokine signaling, and T-cell response.

## Bacterial Pneumonia

Bacterial pathogens are important causes of pneumonia in HCT patients, and a substantial body of work shows the importance of AMs in defending against bacterial infection after HCT (1, 5). AMs isolated from BAL fluid of HCT patients showed diminished chemotaxis, diminished phagocytosis and killing of Candida pseudotropicalis, and diminished killing of Staphylococcus aureus and Listeria monocytogenes (30). Further work in a mouse syngeneic HCT model by Dr. Bethany Moore's group at the University of Michigan has characterized defects in AM function in response to bacterial infection after HCT. Mice receiving syngeneic HCT demonstrated increased bacterial burden, decreased bacterial phagocytosis by AMs, and increased mortality after Pseudomonas aeruginosa intratracheal infection at 21 days post-HCT. The differences in HCT mice occurred despite reconstitution of peripheral blood leukocyte populations and recruitment of leukocytes to the lungs equivalent to control animals (31). Of interest, the defect in phagocytosis of P. aeruginosa by AMs was present in mice that received conditioning *via* TBI as well as those that received conditioning with a combination of cyclophosphamide and busulfan (11).

In investigation of the mechanisms for the AM defects, Ballinger et al. showed that mice receiving syngeneic HCT overproduced PGE_2_ in BAL fluid and in lung homogenates relative to un-transplanted mice (32). Inhibition of PGE_2_ synthesis with indomethacin improved AM phagocytosis of both opsonized and un-opsonized P. aeruginosa, as did the use of a COX-2 knockout mouse as a donor for HCT (11, 32). Further work demonstrated increased vulnerability of syngeneic HCT recipient mice to S. aureus pulmonary infection. Surprisingly, phagocytosis of S. aureus by AMs was actually increased after HCT, but killing of S. aureus was significantly impaired (33).

Further publications from this group have outlined that the mechanism of PGE_2_-dependent reduced phagocytosis and killing by AMs appears to be related to overproduction of prostaglandins and underproduction of leukotrienes (34). Alveolar epithelial cells have an important role in producing PGE_2_ as well as TGF-β in response to cellular stress related to chemoradiation, hypoxia, and free radical injury. In AMs, detection of paracrine TGF-β signaling results in decreased methylation of the COX-2 promoter, leading to further upregulation of PGE_2_ production (35) and signaling through the E prostanoid receptor 2 (EP2). Several downstream effects then occur. Production of the scavenger receptor MARCO decreases and production of the MyD88-dependent IL-1R/TLR inhibitor IRAK-M increases, which together contribute to the decreased phagocytosis of P. aeruginosa (36). PGE_2_ activation also reduces TNF-α and IFN-γ production in AMs from syngeneic HCT mice relative to control (31), while simultaneously increasing production of IL-1β and GM-CSF. This again contributes to a phenotype of impaired AM phagocytosis (37, 38). There are correlations reported in human HCT patients, where elevated AM GM-CSF production and elevated IL-10 have been identified in post-HCT BAL fluid and have been associated with post-HCT lung disease including both suspected and confirmed infections (39). In addition, BAL total TNF-α levels appear higher in infected HCT patients (40). Another effector molecule in the PGE_2_ signaling pathway, Phosphatase and tensin homolog deleted on chromosome 10 (PTEN), was also increased in mouse syngeneic HCT AMs secondary to PGE2 stimulation. Inhibition or knock out of PTEN increased AM phagocytosis and killing of P. aeruginosa with increased production of TNF-α (41). Of note, PTEN appears to inhibit Fc receptor mediated phagocytosis of opsonized bacteria, while IRAK-M inhibits phagocytosis of non-opsonized bacteria (36, 41). These data strongly suggest that PGE_2_ is increased in the lung post-HCT and is responsible for AM dysfunction.

Neutrophils recruited to the lung also have diminished ability to kill P. aeruginosa after syngeneic HCT, though phagocytosis was unchanged (32). Impaired killing by neutrophils is also associated with overexpression of COX-2 and increased PGE_2_ and linked to increased PTEN activity (32, 41) Further investigation has shown that neutrophils recovered from the lung of both allogeneic and syngeneic HCT mice, as well as neutrophils from BALs of human HCT patients, exhibit decreased production of neutrophil extracellular traps (NETs), which are important in antimicrobial defense. The defect in NET production is mediated by PGE_2_ through the receptors EP2 and EP4 (42).

In total, these findings show the critical role of phagocytes, both AMs and neutrophils, in defense against bacterial pneumonia after HCT. The data from syngeneic mouse HCT models implicate TGF-β and PGE_2_ as critical mediators in reducing bacterial phagocytosis and killing. There are also alterations in the inflammatory milieu, with decreased TNF-α and IFN-γ and increased IL-1β and GM-CSF. Human studies have shown analogous data with diminished phagocytic and killing capacity of AMs from allogeneic HCT patients and increased GM-CSF in BAL fluid, although TNF-α was shown to be increased in HCT patients with infection in at least one study.

## Viral Infections

Viruses are also important causes of respiratory infection, whether *via* reactivation of latent viruses such as members of the herpesvirus family or from acute infection such as community-acquired respiratory viruses (1, 43). Cytomegalovirus (CMV) infection remains a significant problem in HCT patients despite antiviral prophylaxis, and other latent viruses in the herpesvirus family such as EBV are also associated with infections and other complications such as post-transplant lymphoproliferative disorder (43). Reflecting this clinical reality, the majority of research on pulmonary immune response to viral infection post HCT has focused on human and murine herpesviruses.

As with IA, genetic polymorphisms in immune-related genes have been associated with susceptibility to viral infection in HCT patients. A donor-derived polymorphism in the TLR9 gene, which is key for recognition of viral PRRs, is associated with decreased risk of CMV pneumonia in allogeneic HCT patients (21). Conversely, both patient- and donor-derived polymorphisms in the IL17A gene were associated with increased risk of CMV infection (29). To date, we have found no investigations of the mechanisms for these polymorphisms to affect susceptibility to viral infection.

BAL samples and lung tissue biopsies from HCT patients have provided important information about the pulmonary antiviral immune response in humans. Milburn et al. showed that allogeneic HCT patients with CMV pneumonitis had lower numbers of lymphocytes, particularly T cells, in BAL fluid relative to patients with pneumonitis caused by other organisms, which may be due to direct cytotoxic activity of CMV against T cells (44). Studies of postmortem lung tissue biopsies from allogeneic HCT CMV patients showed infiltration with both CD4^+^ and CD8^+^ T cells as well as upregulated expression of HLA class II molecules and ICAM-1 on alveolar epithelium suggesting ongoing T cell responses (45). However, another study of lung tissue in HCT patients with infective pneumonitis (primarily CMV) showed increased macrophages and CD4^+^ T cells in the lung, but did not find CD8^+^ T cells or signs of apoptosis (46). The predominant cytokines produced in the lung were associated with Th2 responses, which would cause a polarization away from cytolytic CD8^+^ T cell activation required for viral clearance (46). Bowden et al., however, found an overall increased number of lymphocytes in BAL fluid from HCT patients compared to control, but no correlation between number of CD4^+^, CD8^+^, or CD16^+^ lymphocytes and the presence of pulmonary infection including CMV infection (47). Similarly, Slavin et al. did not find a significant correlation between CMV reactivation and number or percentage of lymphocytes in BAL fluid after HCT (48).

Viral pathogenesis in the lung has also been studied in mouse models. Syngeneic HCT mice infected with murine CMV developed a lymphocytic infiltrate in their lungs composed primarily of CD8^+^ T cells which expressed cytolytic activity *via* recognition of viral peptides (49). An allogeneic HCT mouse model with murine CMV infection also demonstrated increased recruitment of activated CD8^+^ T cells to the lung. The complement regulatory protein decay accelerating factor (DAF) was downregulated in lung tissue, in association with increased serum C3a, in infected HCT mice. Knocking out DAF resulted in worsened survival, increased viral load, increased CD8^+^ T cells in lungs, and increased IFN-γ production in CD8^+^ cells after allogeneic HCT, suggesting an important role for the complement system in defense against viral infection (50). Other studies, however, have shown that functional defects in cytotoxic efficacy of CD8^+^ T cells contribute to CMV infection in HCT mice (51–54). As with pulmonary bacterial infections, the eicosanoid pathway again seems dysregulated in post-HCT pulmonary CMV, with evidence supporting leukotriene deficiency as a mechanism for CMV susceptibility (55).

A mouse gamma herpesvirus, γHV-68, has also been studied in both syngeneic and allogeneic mouse HCT models. Coomes et al. examined the pathophysiology of murine γHV-68 in syngeneic and allogeneic HCT models. Despite a monocyte influx into the lungs, both syngeneic mice and allogeneic HCT mice had impaired response to infection as demonstrated by decreased IFN-γ production as well as increased levels of PGE_2_, T regulatory cells (Tregs), and TGF-β1 in the lungs. The defect in antiviral immunity was shown to be mediated through TGF-β1 (56, 57). Syngeneic HCT mice infected with γHV-68 had T cell responses skewed toward the Th17 phenotype which were associated with increased inflammation and pulmonary fibrosis, as opposed to the Th1 phenotype associated with viral clearance in control mice (58). The Th17 phenotype was associated with the Notch signaling pathway and cellular deficiency of the Notch ligand DLL4 after HCT (58, 59). In addition, migration of conventional DCs 1 and 2 (cDCs 1 and 2) to the lungs was impaired after syngeneic HCT followed by γHV-68 infection, and Th1 cytokine production including IFN-γ were decreased, which together contributed to Th17 skewing (60). The pneumonitis and pulmonary fibrosis seen in this mouse model also resembles the non-infectious lung disease idiopathic pneumonia syndrome (IPS), which is a major complication post-HCT (1, 59).

Similar findings have been detected in murine allogeneic HCT models of herpes simplex virus (HSV) pneumonitis, wherein elevated levels of TGF-β1 in BAL fluid appear associated with the severity of HSV pneumonia (61). Interestingly, levels were highest among transplanted mice with GVHD (which were not receiving any immunosuppressive therapy for GVHD), suggesting a particular propensity toward immune dysregulation and fibrosis. Further, peripheral blood mononuclear cells isolated from HCT patients with HSV infection show natural killer-like phenotype with elevated cytotoxic effects toward human vascular endothelium, which may partly explain the propensity toward bleeding complications in post-HCT viral infections (62).

Community-acquired respiratory viral infections are frequent in HCT patients and can be associated with significant morbidity and mortality (63). The pulmonary immune responses to these viruses after HCT are less well-described. Gowdy et al. examined the effect of the respiratory viral pathogen mouse parainfluenza virus type 1 (mPIV-1), also known as Sendai virus, in both syngeneic and allogeneic HCT mice (64). Allogeneic HCT mice had increased viral load, decreased survival, and increased pulmonary inflammation after Sendai virus infection. This was associated with decreased recruitment of both total and virus specific CD8^+^ T cells to the lungs in allogeneic HCT mice, and was improved by adoptive transfer of CD8^+^ cells from un-transplanted mice recovering from Sendai virus infection. Interestingly, syngeneic HCT mice had similar results to un-transplanted mice after infection (64). In a mouse model of adenovirus infection after allogeneic HCT, McCarthy et al. showed that HCT mice had delayed viral clearance, and although pulmonary PGE_2_ production was increased, the reduced production of IFN-γ and granzyme B and the delayed viral clearing noted in pulmonary CD8^+^ T-cells appeared independent of elevated PGE_2_ production (65).

To summarize, the response to viral pulmonary infections after HCT involves alterations in innate and adaptive immunity. Data on CMV infection in humans demonstrate recruitment of T cells (although there are contradictory findings on involvement of CD4^+^ vs. CD8^+^ subsets) and the presence of Th2 type cytokines in the lungs. Mouse HCT models of murine CMV showed recruitment of but also defects in cytotoxic efficacy of CD8^+^ T cells, as well as involvement of the complement and leukotriene pathways. Similar to mouse models of bacterial pneumonia, TGF-β1in HCT mice infected with γHV-68 mediates diminished antiviral effect and skewing toward Th17 cytokine response. TGF-β1 also appears to be associated with severity of HSV pneumonia in an allogeneic HCT mouse model. Studies of HSV pneumonitis in human HCT recipients suggest cytotoxic effects of leukocytes against vascular endothelium. CD8^+^ T cells were also shown to be essential in defense against two respiratory viruses (Sendai virus and adenovirus) in mouse allogeneic HCT models.

## Discussion

Our review of the literature shows that there are a number of derangements in pulmonary immune responses associated with increased vulnerability to infection after HCT. Reconstitution of AMs in the lung lags behind reconstitution of peripheral leukocytes after HCT, and dendritic cells in the lung are more sensitive to radiation than AMs. The timing of reconstitution of other immune cell types such as T cells in the lung post HCT is unclear. Effective defense against A. fumigatus involves innate immune activation *via* PRRs and post-PRR signaling pathways as well as polarization of the Th cell response toward Th1 and Treg responses and away from Th17 responses. Dendritic cells appear to be key in controlling inflammation and Th response polarization which has a significant effect on the downstream immune response and either control of infection with regulated inflammation or dysregulated inflammation without infection control. This dysregulated inflammation appears to be associated with lung injury and mortality. Clearance of bacterial infection post HCT is compromised by reduced capacity for phagocytosis and bacterial killing by AMs, a phenotype regulated by TGF-β1 which is produced by alveolar epithelial cells and causes increased production of PGE_2_, which in turn mediates defects in AM phagocytosis and killing. Neutrophils are also deficient in bacterial killing and NET production due to PGE_2_ effects. Finally, viral infections require the innate immune system as well as the adaptive immune system for adequate defense, including the activity of CD8+ T cells. The eicosanoid pathway also appears to have an important role in antiviral responses in the lungs post-HCT. Skewing toward Th17 responses involving signaling by TGF-β1 and dendritic cells can have detrimental effects on virus clearance and cause increased inflammatory response and result in a phenotype akin to non-infectious inflammatory HCT-related disorders of the lung such as IPS.

There are limitations to the studies described in this review. Some of the polymorphisms associated with alterations in susceptibility to infections have not had a mechanistic investigation. Studies of HCT patients are limited to BAL fluid and lung tissue biopsies. The mouse HCT models vary in strain, conditioning regimen, method of hematopoietic cell isolation, and dose of hematopoietic cells transplanted, which potentially affect the reconstitution of the immune response. In particular, the majority of the mouse models undergo syngeneic transplants which do not have the alloimmune responses seen in allogeneic human HCT. For example, syngeneic HCT mouse models do not get GVHD and are not receiving immunosuppressive medications for prevention or treatment of GVHD, which is a significant difference from much of the HCT patient population. Beyond this, one must also consider the limitations of mouse models such as genetic differences from humans, lack of underlying disease for which patients undergo HCT, and differences in conditioning and post-HCT treatment.

The lungs have a unique immune response that requires direct sampling for study, as peripheral blood may have different responses. It is particularly important to understand the pulmonary immune response after HCT given the frequency of pulmonary infections and the associated morbidity and mortality despite aggressive prophylaxis and treatment with a variety of antimicrobial medications. Existing studies have identified significant immune changes post-HCT that affect vulnerability to fungal, bacterial, and viral pathogens. New methods such as next generation sequencing can produce large microbiological and transcriptional datasets that can provide large amounts of information about the host response to infection in the lung. Better understanding of these immune changes yields new targets for therapy and may help understand non-infectious lung disease.

## Author Contributions

MZ performed literature review and manuscript writing and editing. JH performed literature review and manuscript writing and editing. All authors contributed to the article and approved the submitted version.

## Conflict of Interest

The authors declare that the research was conducted in the absence of any commercial or financial relationships that could be construed as a potential conflict of interest.
